# *Aplysia* Locomotion: Network and Behavioral Actions of GdFFD, a D-Amino Acid-Containing Neuropeptide

**DOI:** 10.1371/journal.pone.0147335

**Published:** 2016-01-21

**Authors:** Chao-Yu Yang, Ke Yu, Ye Wang, Song-An Chen, Dan-Dan Liu, Zheng-Yang Wang, Yan-Nan Su, Shao-Zhong Yang, Ting-Ting Chen, Itamar Livnat, Ferdinand S. Vilim, Elizabeth C. Cropper, Klaudiusz R. Weiss, Jonathan V. Sweedler, Jian Jing

**Affiliations:** 1 State Key Laboratory of Pharmaceutical Biotechnology, Collaborative Innovation Center of Chemistry for Life Sciences, Jiangsu Engineering Research Center for MicroRNA Biology and Biotechnology, Advanced Institute for Life Sciences, School of Life Sciences, Nanjing University, Nanjing, Jiangsu, China; 2 Beckman Institute for Advanced Science and Technology and Department of Chemistry, University of Illinois at Urbana-Champaign, Urbana, Illinois, United States of America; 3 Department of Neuroscience and Friedman Brain Institute, Icahn School of Medicine at Mount Sinai, New York, New York, United States of America; Texas A&M University - Corpus Christi, UNITED STATES

## Abstract

One emerging principle is that neuromodulators, such as neuropeptides, regulate multiple behaviors, particularly motivated behaviors, e.g., feeding and locomotion. However, how neuromodulators act on multiple neural networks to exert their actions remains poorly understood. These actions depend on the chemical form of the peptide, e.g., an alternation of L- to D- form of an amino acid can endow the peptide with bioactivity, as is the case for the *Aplysia* peptide GdFFD (where dF indicates D-phenylalanine). GdFFD has been shown to act as an extrinsic neuromodulator in the feeding network, while the all L-amino acid form, GFFD, was not bioactive. Given that both GdFFD/GFFD are also present in pedal neurons that mediate locomotion, we sought to determine whether they impact locomotion. We first examined effects of both peptides on isolated ganglia, and monitored fictive programs using the parapedal commissural nerve (PPCN). Indeed, GdFFD was bioactive and GFFD was not. GdFFD increased the frequency with which neural activity was observed in the PPCN. In part, there was an increase in bursting spiking activity that resembled fictive locomotion. Additionally, there was significant activity between bursts. To determine how the peptide-induced activity in the isolated CNS is translated into behavior, we recorded animal movements, and developed a computer program to automatically track the animal and calculate the path of movement and velocity of locomotion. We found that GdFFD significantly reduced locomotion and induced a foot curl. These data suggest that the increase in PPCN activity observed in the isolated CNS during GdFFD application corresponds to a reduction, rather than an increase, in locomotion. In contrast, GFFD had no effect. Thus, our study suggests that GdFFD may act as an intrinsic neuromodulator in the *Aplysia* locomotor network. More generally, our study indicates that physiological and behavioral analyses should be combined to evaluate peptide actions.

## Introduction

Expression of most, if not all, behaviors is regulated by neuromodulators, such as neuropeptides [1]. Indeed, studies in a number of model systems [2–9] have shown that neuromodulators endow behavior with varied and rich characteristics that allow the animal to deal with a variety of environments and achieve diverse goals. To a large extent, prior research entails evaluating actions of neuromodulators in a single neural circuit. However, neuromodulators often affect multiple behaviors [4, 5, 10–13]. Presently, it is poorly understood how a neuromodulator may act in distinct neural circuits to ultimately affect more than one behavior. Here, we took advantage of the well-studied model system, the mollusc *Aplysia* [14–26], to study this issue. Specifically, we sought to examine actions of neuropeptide GdFFD on *Aplysia* locomotor network. GdFFD has been previously shown to act as an extrinsic neuromodulator in the feeding network [27].

GdFFD is of particular interest because it is present in two forms with different bioactivity. Although GFFD, the all L-amino acid form, is expressed during translation, GdFFD is produced through an un-identified isomerase by converting the second amino acid from L-amino acid to D-amino acid. Importantly, this post-translational modification is critical for the function of the peptide because usually the D-amino acid containing isomer confers higher bioactivity than the all L-amino acid form [28]. Indeed, we have shown that in the *Aplysia* feeding circuit [27], only GdFFD is bioactive, while GFFD is not. Intriguingly, relevant to the subject of the current work, we also showed that GdFFD/GFFD are present in neurons of the *Aplysia* pedal ganglia [27] that mediate the locomotor behavior [29–31]. The latter finding raises the possibility that GdFFD and/or GFFD may be bioactive in the *Aplysia* locomotor network. Our test of these peptides in the isolated CNS did show that GdFFD, but not GFFD, induced bursting activity resembling locomotor programs. However, this was accompanied by a significant tonic activity between GdFFD-induced bursts. In the absence of behavioral analyses, it was uncertain whether the tonic activity would be helpful or detrimental to the generation of locomotion. To determine how the enhanced network activity may be translated into locomotor behavior, we designed a behavior recording setup and developed a computer program performing automatic analyses of locomotor path of *Aplysia* that was based on the video recording of *Aplysia* behavior. Although some computer programs analyzing animals’ locomotor behavior are available [32–34], they could not be simply adopted here because of *Aplysia* behavior in an aquatic environment and its distinct body form. Surprisingly, our behavioral analysis suggested that the enhanced network activity corresponded to a reduction, rather than an increase, of locomotion. Our study therefore provides an important example of the necessity of combining physiological and behavioral analyses in the process of evaluating actions of neuromodulators.

## Material and Methods

### Ethics statement

This study was carried out in strict accordance with the Guidelines on the Care and Use of Laboratory Animals issued by the Chinese Council on Animals Research and the Guidelines of Animal Care (Nanjing University, Jiangsu, China), and with the recommendations in the Guide for the Care and Use of Laboratory Animals of the National Institutes of Health (Ichan School of Medicine at Mount Sinai, New York, NY, USA). All surgery was performed under anesthesia by injection of 333 mM isotonic MgCl_2_ (50% of body weight) and all efforts were made to minimize suffering.

### Subjects and reagents

Experiments were performed on *Aplysia californica* (100–250 g) obtained from Marinus, California, USA. *Aplysia* are hermaphroditic (i.e., each animal has reproductive organs normally associated with both male and female sexes). Animals were maintained in circulating artificial seawater (ASW) at 14–16°C and the animal room was equipped with a 24 h light cycle with light period from 6:00 am to 6:00 pm. All chemicals were purchased from Sigma-Aldrich unless otherwise stated.

### *In vitro* electrophysiology

After anesthesia, cerebral and pleural-pedal ganglia were dissected out and maintained in ASW containing the following (in mM): 460 NaCl, 10 KCl, 55 MgCl_2_, 11 CaCl_2_, and 10 HEPES buffer, pH 7.6, in a dish lined with Sylgard (Dow Corning).

Electrophysiological recordings from CNS preparations were performed as described previously [19, 26, 27, 35]. The ventral surfaces of right pedal ganglion and/or left pedal ganglia were desheathed, transferred to a recording chamber containing 1.5 ml of ASW, continuously perfused at 0.3 ml/min, and maintained at 14–17°C. Peptides (GdFFD, UIUC Roy J. Carver Biotechnology Center; GFFD, China-Peptides Inc) were dissolved in ASW immediately before each application, then the ASW with the peptide was perfused into the recording chamber. Before the application of next peptide, it was necessary to perfuse the chamber with only ASW for at least 15 min. Extracellular recordings were made from polyethylene suction electrodes. Extracellular signals were acquired using a differential amplifier Model 1700 (A-M systems). A WPI Pulsemaster Multi-Channel stimulator (SYS-A300) or Grass model S88 stimulator was used for stimulation. Locomotor activity was elicited by brief stimulation of P9 nerve (3–5 s, 3–10 ms, 10 Hz, ~ 0.15 mA or 10–12 Vol). Bursting spiking activity in parapedal commissural nerve (PPCN, or P10) was defined by higher frequency of more than 3 Hz.

### Behavioral analysis

#### (1) Behavior recording setup

Our goal was to automatically analyze video recordings using computer software. In order to obtain accurate data, decrease the time needed for video processing, and to obtain good object-recognition, the experimental environment had to meet the following standards: first, the color or the intensity of the background and the foreground (i.e., the experimental animal and the marker on its body) had to display a large difference; second, the illumination needed to be uniform; third, all the factors that may interfere video recordings, such as obstructions and the reflective surface needed to be minimized. In addition, because of the special characteristics of *Aplysia*, we also took into considerations some extra requirements: first, *Aplysia* live in a cold, aquatic environment, and we kept the water temperature low (i.e. around 14–15°C) and constant. *Aplysia* locomotion may also be influenced by water currents, so the water flow rate was kept relatively low. Second, *Aplysia* sometimes climb on the vertical sides rather than on the bottom, thus, we needed to set the height of the chamber approximately equal to the length of *Aplysia*, so that the vertical movement of *Aplysia* would be limited. Such an arrangement helped the offline analysis of video recordings, which were made from the top of the chamber.

**Fig 1:**
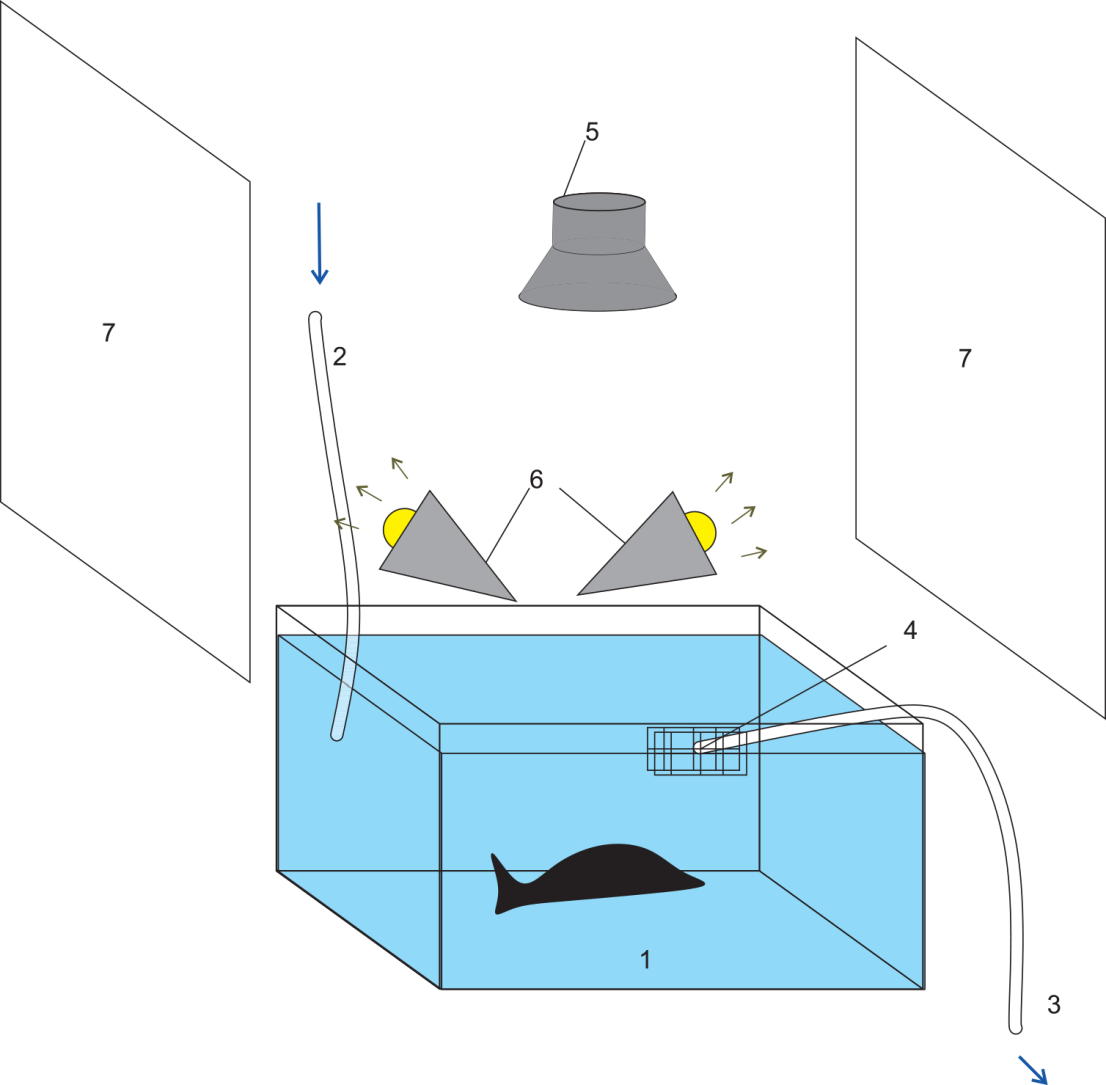
Behavior recording setup. 1. The recording chamber; 2. inlet; 3. outlet; 4. Net (Filter); 5. Camera; 6. Spot light; 7. Reflectors. ASW in the recording chamber is shown in blue for illustration purpose only.

Based on the above requirements, we designed and set up the experimental device (Fig 1). The chamber for studying locomotion was 40×30×18 cm^3^ and was made of white plastic foam. For water circulation, there are an inlet and an outlet. The inlet took seawater directly from a large aquatic tank (~ 400 L) that houses the animal, so that the living environment (i.e. water temperature and salt content) remained unchanged. The flow rate was ~ 1 L/min.

We used a security color camera (16 bit, 640×480 pixel, 12 fps) to record *Aplysia* behavior. The camera was fixed ~ 60 cm directly above the bottom center of the chamber and the focus was on the center of the chamber. The light source was two 12 W spot lights, which illuminated the environment indirectly through two reflectors to prevent the reflection of the light in water.

#### (2) Video recording

Earlier work [36–38] has shown that the activity of *Aplysia* is high and remains relatively constant from 9:00 am to 3:00 pm. Thus, we performed the video recording during this period. At Nanjing University, after *Aplysia* arrival from California, USA, we waited for about a week to perform behavioral experiments to allow *Aplysia* to adapt to the circadian clock in China. First, we placed an animal (100–200 g) in the recording chamber and started the video recording. After the animal adapted to the environment for 30–60 min, we took the animal out and injected it (near its head) with ASW (1 ml per 100 gm animal) or a peptide stock dissolved in ASW. We made a peptide stock at a concentration of 100x the final concentration in the animal, and then injected appropriate amount based on the volume of the animal. For example, a 100 ml animal would be injected with 1 ml of the stock. Next, the animal was gently put back into the chamber and the video recording was restarted after 2 min. The recording was stopped after 1 hour.

#### (3) Analysis of the video

**Fig 2:**
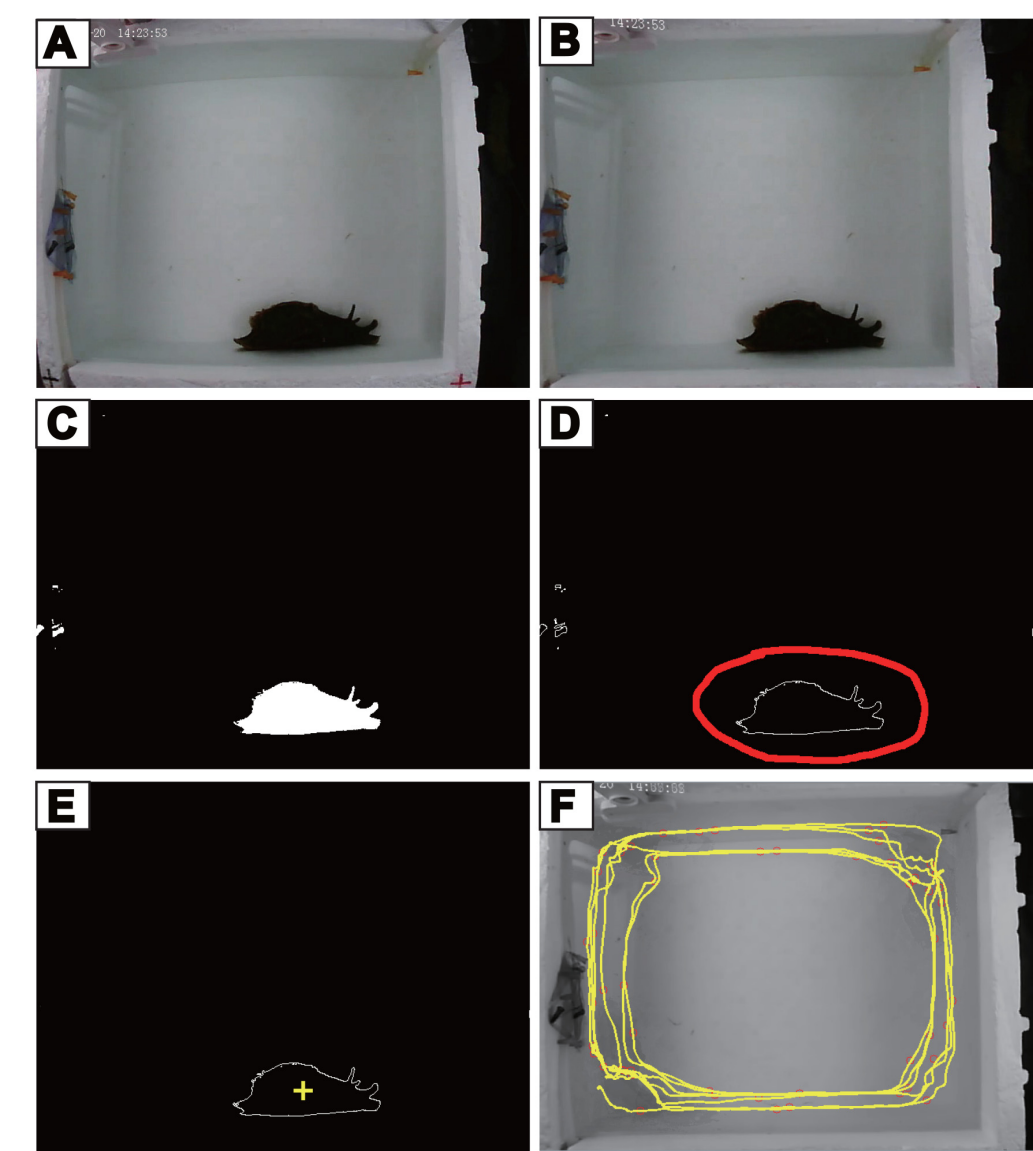
Video analysis procedure. (A) The raw image from a video frame; (B) The image after camera calibration and alignment; (C) The image after color detection and threshold value calculation showing animal outline in white; (D) object-recognition, the object (*Aplysia*) is circled in red line; (E) marking (yellow +) of the object center of gravity; (F) an example of movement path (shown in yellow) of *Aplysia* (1 hr).

All videos were processed through 5 steps as described below (Fig 2). Except for the 1st step (editing and acceleration), the video data were processed by the computer program developed by ourselves. Our program is available as Supporting Information for the paper. Specifically, we provide a manual (S1 Text), the executable files (S1 File) and the source codes (S2 File). The graphing algorithms of our program call up the computer vision library in the widely used open-source software “OpenCV” (http://opencv.org/).

Step 1: editing and acceleration. The goal is to remove the useless data and reduce processing time. This was achieved by using the commercial software “Corel Video Studio” or other film editing software, e.g., Adobe Premiere and the freely available Format Factory (http://www.pcfreetime.com/). Because *Aplysia* move slowly, we reduced the total number of frames of the video to 1/4 of the original, and increased the fame rate from 12fps to 30fps, thus the video play-back was effectively accelerated by 10 times.

Step 2: image calibration and alignment. Because the camera uses a single convex lens, some degrees of tubby (or barrel-shaped) distortions can be formed with a large viewing angle (Fig 2A). By imaging the black and white squares with different distances and importing these pictures to the program with the OpenCV Camera Calibration algorithm, the distortion parameter of the camera was automatically calculated. With the application of “undistort” function (OpenCV) and the distortion parameter in the subsequent analysis, we acquired calibrated coordinates or images (Fig 2B).

During recording, we might have encountered slight video frame movements due to unexpected outside influences. To align the images, we adopted the method of detecting and matching feature points using SURF (Speeded-Up Robust Feature) and FLANN (Fast Library for Approximate Nearest Neighbors) fast matching algorithms and other relevant functions (all from OpenCV). To improve performance, we only calculated one frame in every 100 frames, and used liner interpolation (S2 Text) to complete others.

Step 3: 3-D coordinate model construction. One major difference between locomotion of *Aplysia* and rodents is that *Aplysia* can crawl on the sides of the recording chamber. Although our design of the recording chamber has minimized movements on the vertical sides of the recording chamber, such movements are present and cannot be ignored. We want to emphasize, however, that unlike some *Aplysia* species, *Aplysia californica* do not swim; they crawl on substrate. We constructed a 3-D coordinate-based model consisting of three-dimensional space (i.e. the x, y, z coordinates). We projected one bottom and four side surfaces into one plane. The corresponding coordinates of the points in the chamber to one plane were acquired by importing and calculating the image coordinates of the angular points and the distances between different surfaces.

Step 4: object (*Aplysia*) recognition. To recognize and track the animal and calculate its position, we used the following procedures: a). Extracting the background based on the color difference between background and the animal using BackgroundSubtractorMOG2 algorithm of OpenCV; b). Color matching: obtain color histogram of the animal, and localize the area in the video frame similar to animal color, to obtain object outline using Threshold function (OpenCV); c). Producing object outline based on animal color using findContour function (OpenCV) (Fig 2C and 2D), while excluding the objects (e.g., feces) that may have similar color to the animal based on different sizes and other characteristics. d). Calculating the center of gravity of the animal (Fig 2E), and generating the coordinates of the animal in the 3-D space. During the execution of the program, a command line option "-m" (S1 Text) enables a pop-up window which displays the recognized animal in each video frame as the program is processing the video. Any problem with recognition can be visualized and remedied by adjusting a parameter (S1 Text).

Due to unintended camera interruption and other factors, position data of the recognized object in a frame may be invalid. We validated object detection in each frame, and if deemed to be invalid, we ignored the frame(s), supplemented the lost data using liner interpolation (S2 Text).

Step 5: locomotor path and velocity. After arraying the coordinates in order of time, we obtained *Aplysia* locomotor path. To deal with potential noise (within a few pixels) due to random factors, which could lead to the calculated distance being longer than the actual, we removed the noise using the average filtering method implemented by us as described in supporting information (S3 Text). Finally, we calculated the average velocity, *v* (mm⋅h^−1^), of *Aplysia* locomotion with the calibrated distance (Fig 2F) divided by the time.

**Fig 3:**
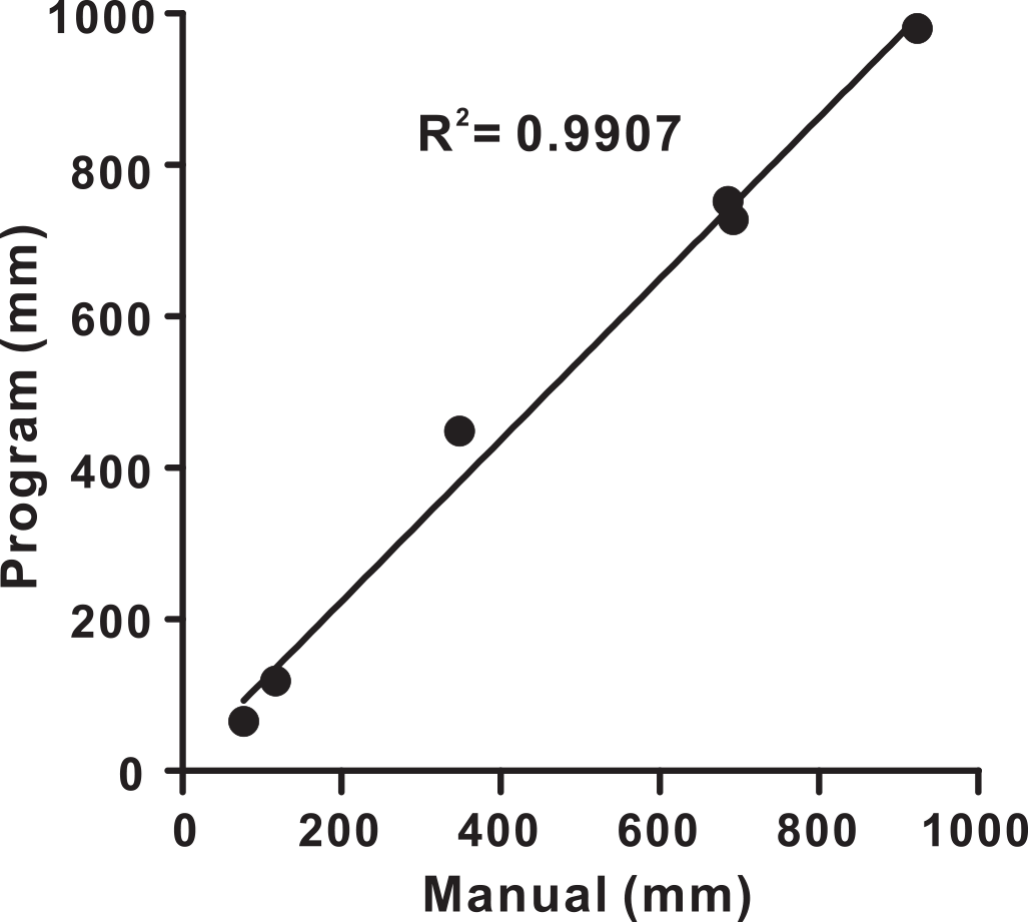
Validation of the program. Plot of the paths (in mm) that animals traveled in six video segments determined both manually and automatically by the program. The thick line is the linear regression fit. The two sets of path data were highly correlated with a slope of 1.066.

We validated the program by selecting six 10-min video segments from six animals. The video segments were reduced and sped-up, so there were 1800 frames per segment. We used our program to quantify total paths from processed videos. We then manually calculated the path that the animal traveled. We reduced the work load to a reasonable amount by selecting 1 frame per 150 frames (a total of 13 frames for each segment). We performed linear regressions on the path data obtained manually and through the program (Fig 3). The two sets of path data are highly correlated (r^2^ = 0.9907, p < 0.0001) with a slope of 1.066, indicating that the data from both methods were similar. The average data from the manual calculation (473 ± 141 mm) were somewhat smaller than those from the program (515 ± 151 mm). This is not unexpected. Manual calculations may miss transient movements as a result of longer inter-frame intervals.

We determined if we could use *v* as a parameter for statistical comparison of whether ASW or peptide injection can impact locomotion. We found that the average velocity from ten control animals before ASW injection did not pass the Normality Test (Shapiro-Wilk, which compares the deviation of the data with deviation expected in a data set with normal distribution, p < 0.05, Sigmaplot 12.5). We then utilized the commonly-used natural logarithm of *v* to define *u* (i.e., *u* = ln *v*), and found that *u* from the ten control animals did pass the Normality Test (Shapiro-Wilk, p > 0.05). We therefore chose *u* as the parameter to represent the activity level and to perform statistical analyses.

### Additional data analysis

Electrophysiological recordings were digitized online with Axoscope (Molecular Devices) and plotted with CorelDraw (Corel). Bar graphs were plotted using SigmaPlot. Data are expressed as mean ± SEM. Statistical tests included Student’s t-test and repeated-measures one-way ANOVA, and were applied as appropriate using Prism software (GraphPad) unless otherwise stated. Data that showed significant effects in ANOVA were further analyzed in individual comparisons with Bonferroni’s correction. In all statistical tests, effects were considered statistically significant when p < 0.05.

## Results

### Effects of GFFD and GdFFD in the isolated CNS of *Aplysia*

**Fig 4:**
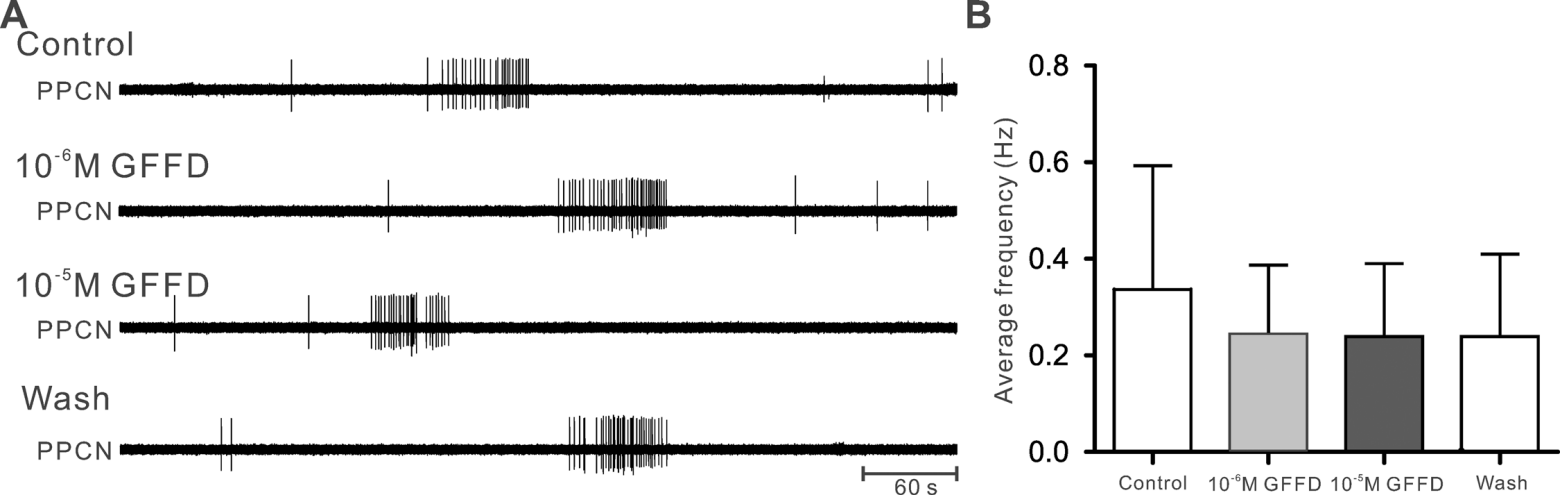
Absence of effects of GFFD in the isolated cerebral and pleural-pedal ganglia. (A) The electrophysiological recordings of PPCN during control, after perfusion with 10^−6^ M or 10^−5^ M GFFD, and during wash, each recording lasting ~ 10 min. (B) Group data of the average PPCN frequency over 10 min under different conditions. GFFD had no significant effects on PPCN activity. Error bars indicate SEM.

**Fig 5:**
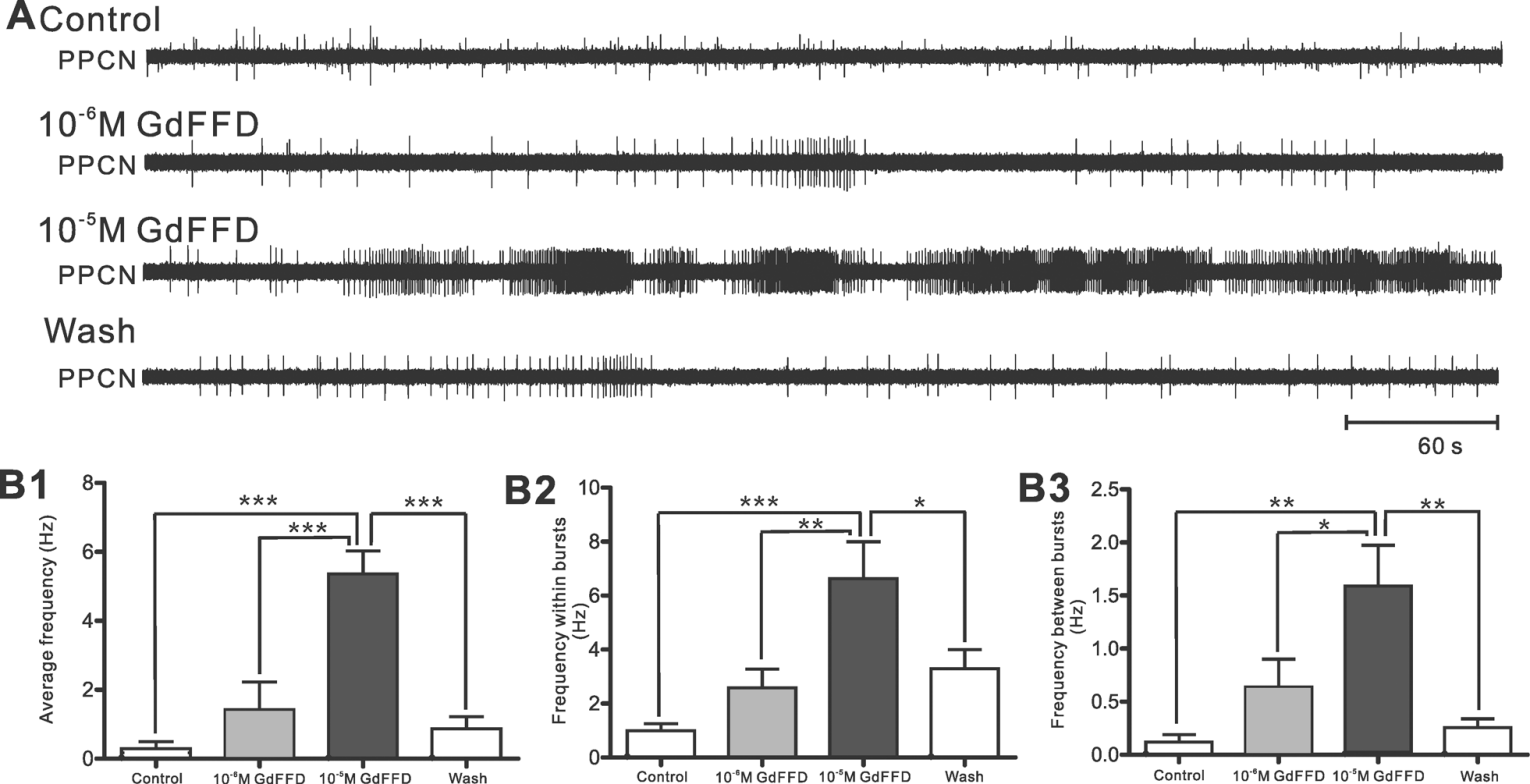
GdFFD induces activity in the isolated cerebral and pleural-pedal ganglia. (A) The electrophysiological recordings of PPCN during control, after perfusion with 10^−6^ M or 10^−5^ M GdFFD, and during wash, each recording lasting ~ 10 min. (B) Group data. (B1) The average frequency of PPCN over 10 min; (B2) The average of frequency within bursts in PPCN over a period of 10 min; (B3) The average of frequency between bursts in PPCN over 10 min. Bonferroni post hoc tests: *p < 0.05; **p < 0.01; ***p < 0.001. Error bars indicate SEM.

**Fig 6:**
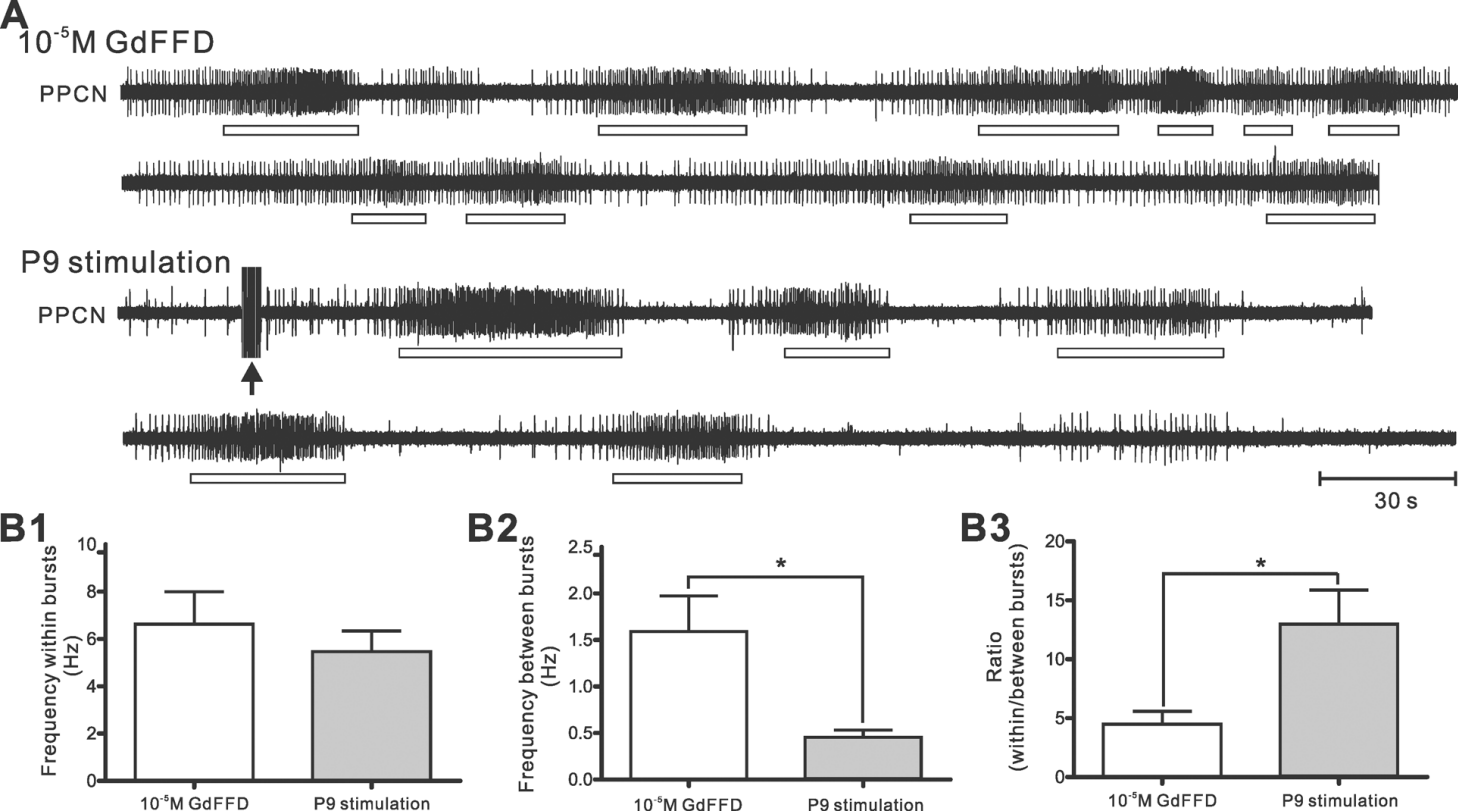
Comparison between network activity induced by GdFFD perfusion and by P9 stimulation. (A) The electrophysiological recordings of PPCN during 10^−5^ M GdFFD perfusion or after P9 stimulation. The two traces in each condition are continuous. The black arrow marks P9 stimulation. Both GdFFD perfusion and P9 stimulation induced bursting activity, but activity between bursts was higher with GdFFD perfusion. Open bars indicate PPCN bursting activity. (B) Group data. (B1) The average of frequency within bursts of PPCN over 10 min; (B2) The average of frequency between bursts of PPCN over 10 min. (B3) The ratio of frequency within bursts to frequency between bursts. Paired two-tailed t-test: *p < 0.05; Error bars indicate SEM.

Although previous work in *Aplysia* feeding network [27] indicated that only GdFFD was bioactive, a priori we did not know if the same holds true in the locomotor network. Thus, one major goal of the present work was to examine potential bioactivity of both GFFD and GdFFD on the locomotor network. To achieve this, we isolated the cerebral and pleural-pedal ganglia and sought to examine how GFFD and GdFFD may impact activity of the locomotor network in these ganglia. Locomotor behavior is mediated by a series of rhythmic contractions of foot and body muscles moving from the anterior part of the body to the posterior part, called pedal waves [39]. Previous work has shown that pedal ganglia contain the central pattern generator for locomotion [29–31], and the cerebral ganglion contains command-like and modulatory neurons that can influence the expression of locomotor activity [40, 41]. The locomotor activity was monitored by parapedal commissural nerve (PPCN or P10) that innervates the posterior part of the foot, and was shown previously to exhibit bursting activity in phase with locomotion, and apparently represents motoneuronal activity [29–31, 41–43] (Figs 4A, 5A and 6A).

In each experiment, we recorded baseline activity of the cerebral and pleural-pedal ganglia for more than 15 min, then we perfused 10^−6^ M peptide for more than 15 min, and followed this by 10^−5^ M peptide perfusion for more than 15 min. Then peptide was washed out with ASW for 20 min or until the effect disappeared, whichever was longer. We found that perfusion of GFFD at 10^−6^ M or 10^−5^ M did not change the activity in PPCN (Fig 4A). Group data showed that there was no significant change in PPCN activity with perfusion of GFFD (Fig 4B, PPCN average frequency over 10 min, Control: 0.34 ± 0.25 Hz; 10^−6^ M: 0.24 ± 0.14 Hz; 10^−5^ M: 0.24 ± 0.15 Hz; Wash: 0.24 ± 0.17 Hz; F_3,9_ = 0.8498; p > 0.05; n = 4).

In contrast, perfusion of GdFFD at 10^−6^ M, and more pronouncedly at 10^−5^ M, increased activity in PPCN (Fig 5A). In particular, during perfusion of 10^−5^ M GdFFD, the PPCN showed persistent bursting activity resembling locomotor activity that lasted for as long as the peptide was present. Surprisingly, we also observed apparent increase in PPCN activity between bursts, although at lower frequency. Group data showed that GdFFD perfusion significantly increased PPCN activity that was reflected in the average frequency (Fig 5B1, Control: 0.29 ± 0.2 Hz; 10^−6^ M: 1.41 ± 0.81 Hz; 10^−5^ M: 5.36 ± 0.68 Hz; Wash: 0.86 ± 0.35 Hz; F_3,12_ = 26.33; p < 0.001; n = 5), frequency within bursts (Fig 5B2, Control: 0.99 ± 0.26 Hz; 10^−6^ M: 2.58 ± 0.7 Hz; 10^−5^ M: 6.63 ± 1.37 Hz; Wash: 3.28 ± 0.72 Hz; F_3,12_ = 14.77; p < 0.001; n = 5), and frequency between bursts (Fig 5B3, Control: 0.12 ± 0.07 Hz; 10^−6^ M: 0.64 ± 0.26 Hz; 10^−5^ M: 1.59 ± 0.38 Hz; Wash: 0.25 ± 0.08 Hz; F_3,12_ = 10.16; p < 0.01; n = 5).

Thus, the above data indicate that, similar to their effects on the feeding network, GdFFD, but not GFFD, is bioactive in the locomotor network.

### Comparison of GdFFD-induced PPCN activity with P9 stimulation-induced PPCN activity

Although the effects of GdFFD on the cerebral and pleural-pedal ganglia are apparent, it is uncertain whether and how it would impact locomotor programs. As a first step toward understanding what the increased PPCN activity during GdFFD perfusion may represent, we compared GdFFD-induced PPCN activity with P9 stimulation-induced PPCN activity (Fig 6A). P9 is a mixed sensory-motor nerve that carries sensory information from the tail, and brief stimulation of the tail or P9 has been shown to reliably induce locomotor activity in both intact animals and the isolated CNS [29–31, 41, 43]. Fig 6A shows an example of PPCN activity induced by GdFFD (at 10^−5^ M) and P9 stimulation in the same preparation. In both cases, there was bursting activity in PPCN. However, there was clear activity between bursts during GdFFD perfusion, that was largely absent in P9 stimulation-induced PPCN activity. Group data showed that PPCN frequency within bursts during GdFFD perfusion was somewhat higher than after P9 stimulation, but did not differ statistically (Fig 6B1, GdFFD: 6.63 ± 1.37 Hz; P9 stimulation: 5.47 ± 0.87 Hz; p > 0.05; Paired two tailed t-test, n = 5). However, PPCN frequency between bursts (Fig 6B2, GdFFD: 1.59 ± 0.38 Hz; P9 stimulation: 0.46 ± 0.07 Hz; p < 0.05; Paired two tailed t-test, n = 5) was higher during GdFFD perfusion than after P9 stimulation. This suggests that during GdFFD perfusion, although there was bursting activity, the tonic activity between bursts might reduce rhythmicity of PPCN bursts. To illustrate this point, consider a case, in which activity between bursts increases to the level that is high enough to approach the level of frequency within bursts. In such a case, the overall activity would be simply tonic. Thus, we calculated the ratio of frequency within bursts over frequency between bursts, and a lower ratio would represent a lower rhythmicity. Indeed, we found that the ratio was lower during GdFFD perfusion than after P9 stimulation (Fig 6B3, GdFFD: 4.48 ± 0.59 Hz; P9 stimulation: 12.98 ± 2.89 Hz; p < 0.05; Paired two tailed t-test, n = 5).

### Video analysis of *Aplysia* behavior

The above analysis clearly showed that GdFFD-induced PPCN activity differs from P9 stimulation-induced activity with higher activity between bursts. In terms of how the GdFFD-induced activity may be translated into behavior, in theory, we are still left with two possibilities. One is that the bursting PPCN activity induced by GdFFD may support higher locomotor activity, although the locomotor movements may differ somewhat from normal locomotion either spontaneously present or induced by noxious stimulation (e.g., to tail). Another possibility is that excessive activity in motor nerves (e.g., PPCN) between bursts may prevent the animals from performing normal locomotion. The only way to determine conclusively which possibility is correct is to perform behavioral experiments and observe *Aplysia* behavior. Specifically, we tracked *Aplysia* in a behavior recording setup before and after injection with ASW or different concentrations of GdFFD or GFFD. To analyze the video recording data, we developed a video analysis program to automatically detect *Aplysia* in the video frames and track its path, and to calculate the velocity of *Aplysia* locomotion.

**Fig 7:**
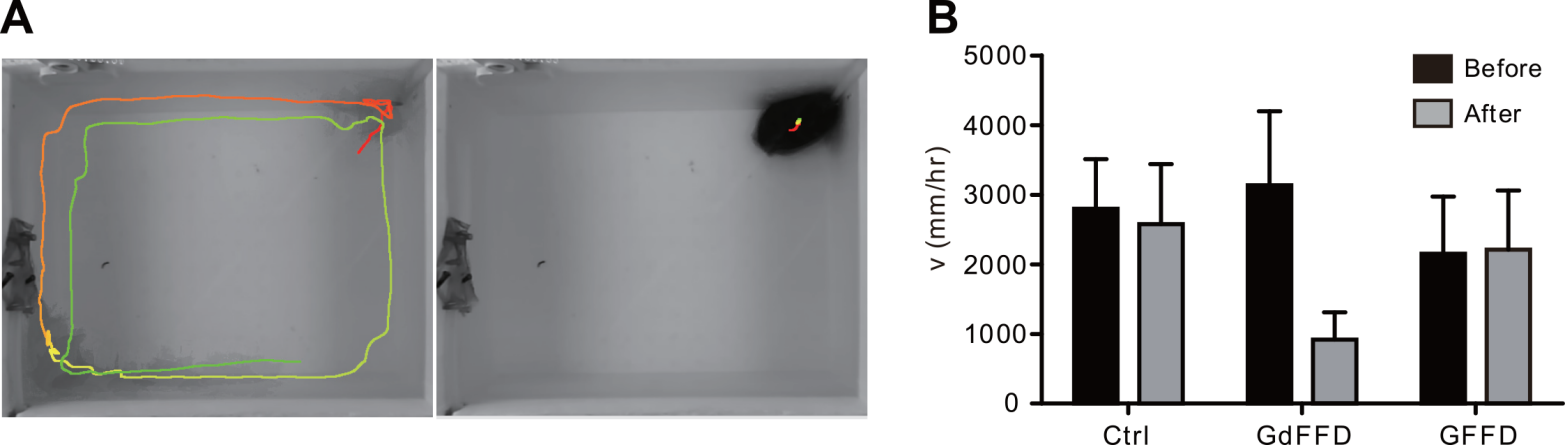
GdFFD reduces *Aplysia* locomotion whereas GFFD has no effects based on video analysis. (A) The locomotor path of *Aplysia* after injection with ASW or GdFFD. Left panel: ASW; right panel: 10^−8^ M GdFFD (The animal is present at top right corner as it typically stays in the area). Color of path: red: beginning of path; blue: end of the path. (B) Group data. The velocity (v) before (black bars) and after (gray bars) injections with ASW, 10^−8^ M GdFFD or 10^−6^ M GFFD. Error bars indicate SEM.

For each experiment, we removed one animal from a tank housing the animal, put it in the behavior recording chamber, and recorded the animal for 60 min. Then we took the animal out, injected either ASW (control), GFFD or GdFFD, put it back into the behavior recording chamber, and recorded it again for 60 min. We observed no change in *Aplysia* behavior after injection of ASW (Control: 2800 ± 700 mm/hr vs. After ASW injection: 2600 ± 900 mm/hr) (Fig 7A-left panel and 7B) or GFFD (Control: 2200 ± 800 mm/hr vs. GFFD: 2200 ± 800 mm/hr). However, after GdFFD was injected, we observed that the foot of *Aplysia* curled up, and *Aplysia* seemed to have difficulty to wriggle and adhere to the side wall or substrate. With a higher concentration of GdFFD, even though the head and tentacles can move normally, *Aplysia* were observed to open its operculum (gill cover), and the parapodia showed some peristaltic waves. Overall, *Aplysia* typically stayed around the same place for long periods of time and showed little normal locomotor movements (Fig 7A-right panel). Thus, the distance the animals traveled during video recording decreased compared with the control (Control: 3100 ± 1100 mm/hr vs. GdFFD: 900 ± 400 mm/hr) (Fig 7B).

For our statistical analysis, as described in Methods (“Behavioral analysis” section), we did not use the locomotion velocity (*v*). Instead, we used its transform *u* (i.e., *u* = ln *v*) because this parameter passed Normality Test (Shapiro-Wilk). For the control experiments, we found that there was no significant difference when we compared *u* before injection of ASW and after injection of ASW (Before injection: 7.71 ± 0.21; After injection: 7.52 ± 0.26; Paired two-tailed t-test, p > 0.05, n = 10). The data suggests that injection by itself did not influence activity level of *Aplysia*.

For the peptide experiments, initial test of GdFFD with different concentrations showed that 10^−8^ M GdFFD already had obvious effects. We also tested a higher concentration, i.e., 10^−7^ M, of GdFFD, and found that it had much stronger inhibitory effects. Thus, we used 10^−8^ M in all the rest of the experiments (n = 4). Note that the concentrations used in intact animals and the isolated CNS were not comparable. In intact animals, peptides presumably migrate into the CNS through active vascular circulation, whereas in the isolated CNS, peptides migrate into the CNS (especially the neuropile) through passive diffusion. Consequently; it is not surprising that a lower peptide concentration is needed in intact animals. We found that, at 10^−8^ M GdFFD, there was a significant difference compared with the control (*u* value, Before injection: 7.89 ± 0.32; After injection: 6.49 ± 0.51; Paired two-tailed t-test, p < 0.05), i.e., GdFFD significantly reduced locomotion.

Initial test of GFFD with different concentrations (10^−8^ M and 10^−7^ M) showed no obvious effect, so we selected a higher concentration, 10^−6^ M GFFD (n = 4) for the statistical test. Even with this concentration, there was no significant difference compared with the control (*u* value, Before injection: 7.46 ± 0.37; After injection: 7.44 ± 0.43; Paired two-tailed t-test, p > 0.05).

In summary, GFFD appeared to have no significant effects on locomotor activity in the isolated CNS and intact animals. In contrast, GdFFD increased nerve activity in the isolated CNS, and translated into behavior, its effect was a significant reduction in locomotion.

## Discussion

In the present study, we characterized the actions of GdFFD on *Aplysia* locomotor network both in the isolated CNS and in intact animals. Together with the previous work [27], our findings indicate that GdFFD acts in two distinct neural circuits to affect the expression of two behaviors, i.e., feeding and locomotion.

### Video analysis of behavior using computer programs

In the course of the study, we realized that the effects of GdFFD on locomotor programs in the isolated CNS can not unequivocally be translated into an effect on locomotor behavior. Thus, physiological analysis of GdFFD effects in the isolated CNS was insufficient, and it was necessary to evaluate directly the effects of GdFFD/GFFD on behavior. For this, we first designed a behavior recording setup that allows *Aplysia* to move freely in a temperature-controlled chamber. We video-recorded *Aplysia* behavior with a camera for 30 min or longer. We developed a video analysis program to track animal locomotor path and automatically calculate its route.

Currently, significant progress has been made toward automatic video analysis of behavior using computer programs, and some systems are available commercially. Notably, Wolfer et al [32] developed a public domain software Wintrack to perform numerical and graphical path analysis of mice locomotor movements tracked using EthoVision. There are also OpenControl [33] and ETHOWATCHER [34] systems applied to rats. These two systems can automatically track rats’ position, draw their locomotor path, and perform statistical analysis, e.g., movement rate, direction and pausing time. Video analysis systems have also been made for lobster [44] and *Drosophila* [45]. Unfortunately, we cannot directly adopt the above systems for our work in *Aplysia*. First, *Aplysia* prefer to crawl on or close to the sides of the tank wall, thus their movements are not confined to one plane. Second, *Aplysia* bodies are soft, and their shape can change significantly during movement, thus their bodies are difficult to recognize solely based on shape. Finally, although video analysis of behavior in *Aplysia* has also been described [38, 46, 47], these studies did not perform automatic analyses of *Aplysia* locomotor path, thus necessitating the development of our software system.

The application of our newly developed computer program to study the actions of neuropeptides on locomotor behavior clearly demonstrated the utility of our system. The software quantifies *Aplysia* behavior, in particular, it calculates the locomotor path. Specifically, from video recordings of *Aplysia* obtained from our specifically-designed behavior recording setup, the program corrects distorted images, recognizes the animal, tracks its path, and calculates locomotor velocity within a specified time period. Our analysis showed that the program is robust. First, the detection of *Aplysia* in the video frames is resistant to various interferences, including brief interruption of video recording, and appearance of undesired objects in the video scene; second, video frame shift due to minor camera movement can also be corrected automatically. Indeed, our calculation of locomotor path proved to be quite accurate, and the path data with high precision were produced. Thus, our program not only makes it easier/faster to obtain the path data of *Aplysia* locomotion, it also eliminates the human error/bias that can arise when the path data are obtained manually.

The program has room for improvement. For example, we would like to speed up the running time of software. Currently, it took about 10 min to analyze 6 min video at 30 f/s. In addition, functions of the program are somewhat limited compared with some other available systems. Specifically, we would like to develop a more user-friendly interface. Despite some limitations, the program possesses all the necessary functions to automatically analyze locomotor path of *Aplysia* from video recordings in a behavioral chamber.

### Modulatory actions of GdFFD on feeding and locomotion

Previously [27], we have characterized, for the first time, the actions of a D-amino acid containing peptide in a well-defined circuit. Specifically, we showed that only GdFFD, not GFFD, activates egestive type of motor programs in the *Aplysia* feeding circuit. Interestingly, although GdFFD/GFFD are not present in the cell bodies of neurons of the buccal ganglion that contains the feeding central pattern generator, we did find that they are present in the processes in the buccal ganglion which apparently originate from GdFFD/GFFD positive neurons elsewhere (e.g., the pedal ganglion). Thus, GdFFD acts as an extrinsic modulator in *Aplysia* feeding network.

In this paper, we showed that GdFFD also acts in *Aplysia* locomotor network, although likely as an intrinsic modulator because GdFFD positive neurons are present in the pedal ganglia. In the isolated CNS, GdFFD, but not GFFD, enhances the network activity as represented by PPCN activity by inducing bursting resembling locomotor programs. However, it also increased activity between bursts. There are two possible ways to interpret these *in vitro* effects. First, the enhanced bursting activity in PPCN might suggest an enhanced locomotor activity. Second, the significant activity between bursts induced by GdFFD differs from nerve P9 stimulation-induced PPCN activity with much weaker activity between bursts. This observation may therefore suggest a different behavioral effect, i.e., potentially interfering with locomotion. The only way to resolve this ambiguity is to examine directly what GdFFD might do to locomotor behavior. We therefore performed behavioral experiments and used our computer program to quantify the locomotor path of *Aplysia* in the absence and the presence of peptides, i.e., GFFD/GdFFD. We found that only GdFFD is bioactive, while GFFD is not, consistent with results obtained from the isolated CNS preparations. To our knowledge, the findings in both Aplysia feeding [27] and locomotor circuits may be one of the first demonstrations that a D-amino acid in a peptide is required to be bioactive in two distinct circuits (see also [48]). Moreover, we found that GdFFD actually reduces, rather than increases locomotor activity. Indeed, in the presence of GdFFD, *Aplysia* maintained a curled posture, apparently unable to move, which might well be caused, at least in part, by too much overall, rather than rhythmic, motoneuronal activity (i.e., PPCN).

Thus, one of most significant implications of the present work is that evaluation of actions of neuromodulators in neural circuits should involve behavioral experiments. This is especially true in cases where the neuromodulator-induced activity in the isolated CNS differs from spontaneous or nerve-induced activity, and thus the behavioral meaning of the activity *in vitro* is ambiguous. Over the years, a large number of studies in several model systems [2, 5, 8, 9] investigated how neuromodulators affect various CPGs. Although some attempts to directly observe behavioral effects of modulators have been made [49–53], for the most part, the work has been focused on *in vitro* preparations. For behavioral studies, *Aplysia* locomotion is well suited for automatic analyses using computer programs. Thus, our study offers an excellent example for which a combined physiological and behavioral approach may be fruitful.

Another important implication of our work is that we clearly showed that the same neuropeptides can exert significant actions in multiple neural circuits, i.e., feeding and locomotion. Both feeding and locomotion are motivated behaviors as their expression is under influence of a variety of intrinsic factors, including neuromodulators [2–9]. For example, in *Aplysia*, feeding can be modulated by serotonin and a number of peptides. *Aplysia* locomotion is also modulated by serotonin [41, 54], whereas the neuropeptides that act on locomotion are less well understood [55–57]. Currently, among the known neuromodulators, perhaps the actions of serotonin are the best studied, and serotonergic neurons in feeding, locomotion, and withdrawal act in concert to contribute to the establishment of an arousal state [5, 16]. In terms of GdFFD, it remains to be determined what state GdFFD may contribute to. Interestingly, GdFFD induces motor activity in both feeding and locomotor networks. In feeding, GdFFD appears to promote egestion [27], whereas for locomotion, it promotes a curled up posture and actually reduces locomotion. Perhaps these effects would contribute to the establishment of a withdrawal state in which *Aplysia* reject food, and then stay in a confined area, possibly to avoid predators. In the future, it would be of interest to determine which GdFFD-containing neurons are responsible for these actions and under what conditions these GdFFD-containing neurons could be active. The answer to these questions would give us a better understanding of what behavioral state GdFFD might contribute to. In addition to the tonic effects of the exogenous GdFFD demonstrated in this paper, alternative modes of GdFFD actions may also occur. First, normal release of the neuropeptide may occur from GdFFD containing neurons that show a rhythmic pattern of activity, thus resulting in phasic actions of GdFFD. Second, GdFFD may act in conjunction with other neuromodulators. In either case, GdFFD may potentially promote locomotion rather than reducing locomotion.

In a more general sense, this work provides evidence that post-translational isomerization from an L- to a D-amino acid in a neuropeptide, where it exists, is usually required for the bioactivity of a peptide likely because the isomerized peptide may bind to a receptor with higher affinity than its all-L-amino acid epimer [28, 58–63].

## Conclusions

In summary, using both physiological and behavioral approaches, we have successfully characterized actions of GdFFD/GFFD on *Aplysia* locomotor network. Overall, our examination of actions of GdFFD and GFFD in *Aplysia* feeding [27] and locomotor networks showed that only GdFFD is bioactive in the two distinct circuits, reinforcing the importance of post-translational modification to D-amino acid containing peptide for the functioning of the peptide. Moreover, we showed that *in vitro* characterization of peptide actions may not be sufficient at least in some cases. Rather, behavioral assessment of peptide actions may be necessary for a complete understanding.

## Supporting Information

S1 Text Program manual: A manual for using our program to analyze *Aplysia* locomotion. (PDF)

S2 Text Validation of object detection and interpolation. (PDF)

S3 Text Average filtering. (PDF)

S1 File Executable files: A compressed file containing all compiled files necessary to run the program. (7Z)

S2 File Program source code: A compressed file containing files for all the source codes. (7Z)
