# Supplementary material for: Aplysia Locomotion: Network and Behavioral Actions of GdFFD, a D-Amino Acid-Containing Neuropeptide
Source: PLoS One. 2016 Jan 21;11(1):e0147335. doi: 10.1371/journal.pone.0147335 (PMC4721866; doi:10.1371/journal.pone.0147335)
Supplement: S1 Text — (PDF) [file pone.0147335.s001.pdf]

Aplysia Behavior Analysis Tool Manual

1. Introduction

This tool is designed to analyze the locomotion behavior of *Aplysia californica*, and is potentially adaptable to behavior analysis tasks involving other animals.

Files included: 1) Executable files: A compressed file (**S2_File.7z**) containing all compiled files necessary to run the program. Uncompress **S2_File.7z**, and follow instruction 2-4 to run the program. 2) Program source code: A compressed file (**S3_File.7z**) containing files for all the source codes. Uncompress **S3_File.7z**, and examine and/or modify the source codes. Follow the instruction 6 (Appendix) to compile the codes. Please inform the author (poooon@foxmail.com) if you would like to make any improvements.

2. Execution

To run the tool, execute the file **analyzer.exe** in the **analyzer** folder through command line (To run command line in Windows 7, go to **Windows Menu** -> **Run** -> type in **"cmd"** and click **run**). For example, if the **analyzer.exe** is in the directory "D:\research\build\analyzer\", you can type in the following commands in the command line window:

```
>D:
>cd research\build\analyzer
>analyzer -a "D:\research\align\sample.a.txt" -o
"D:\research\data\sample.o.txt" -m -b "calibration.txt"
"D:\research\video\sample.mp4"
```

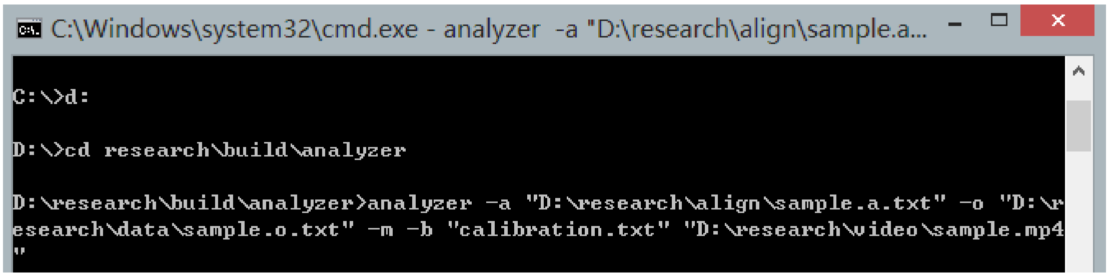

Add the following command line options to properly use this program, put a space between each option.

"`-a "D:\research\align\sample.a.txt"`" to use the file "D:\research\align\sample.a.txt" as the align file. Align files are calculated based on each video file. If there is no existing align file in that name, a new one will be generated. Use simply "`-a`" to always create a new align file.

"`-o "D:\research\data\sample.o.txt"`" to output the results to the

file "D:\research\data\sample.o.txt". If the designated file already exists, it will be rewritten with new results.

"-m" to monitor the detection of process in a new window showing each frame of the video and the detected path of the animal.

"-b "calibration.txt"" to use "calibration.txt", which should be in the **analyzer** folder, as the camera calibration file. A calibration file helps to correct, if any, your camera's distortion. To create a calibration file for your camera, check:

http://docs.opencv.org/2.4.11/doc/tutorials/calib3d/camera_calibration/camera_calibration.html.

""D:\research\video\sample.mp4"" to point to the target video file. You always need to add this option as the last of command line option.

In massive processing, it is recommended to use shell scripts rather than using command line input directly. For example, you can write a MS-DOS script like this:

```
@echo off

set apppath=D:\research\build\analyzer\
set executable=%apppath%analyzer.exe
set videodir=D:\research\video\
@echo on

cd %apppath%
for %%f in (%videodir%*.mp4) do %executable% -a %%f.a.txt
-b "calibration.txt" -o %%f.o.txt %%f
```

Save it as "xxx.bat", then run it through command line. All mp4 video will be processed, and the results will be put under the same directory with the videos.

3. Processing

When you are running the program with "-m" option, a detection pop-up window will show:

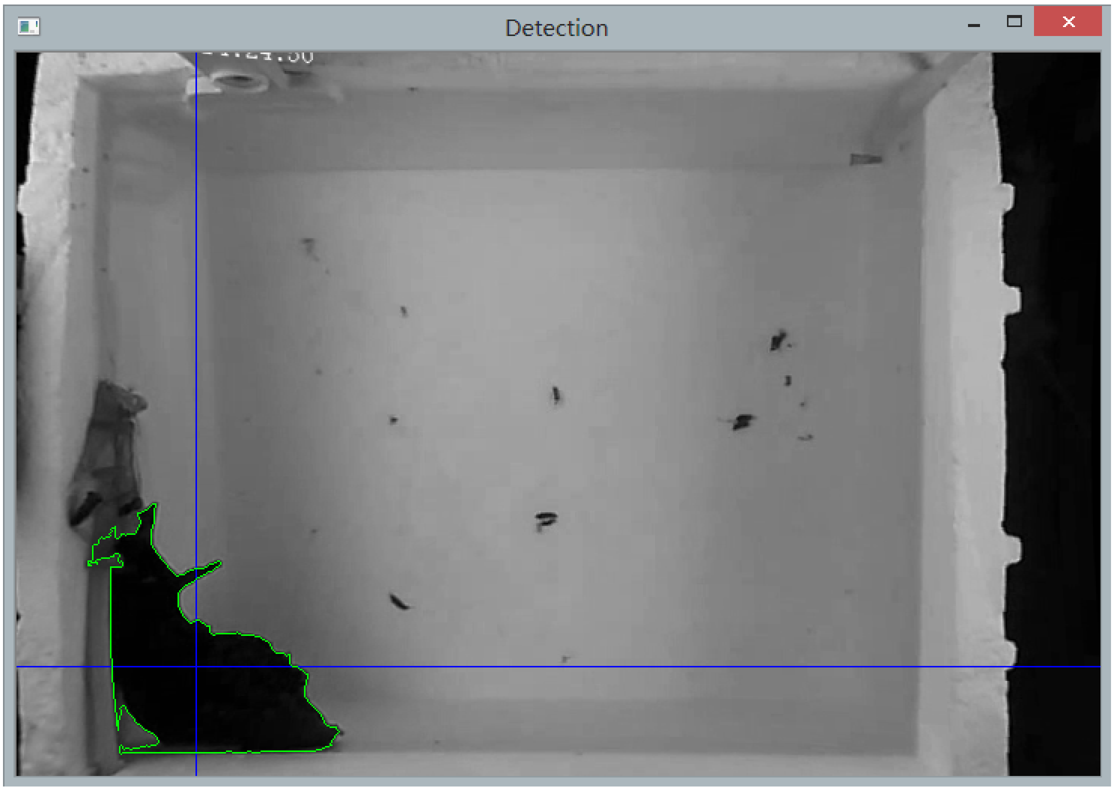

You can check if the cross is focusing the center of animal's body, and if the green line properly fits the animal's contour. **You should always check the detection process as described above to ensure the validity of your data**. If the cross focuses on some other objects than the animal, or the detected contour is often too large or too small to fit the animal, you need to adjust the parameters in the configuration file **config.txt** in the folder to find a proper value. Try a lower **threshold** value if wrong objects are detected, and a higher value if the animal is not wholly circled in. Save the modified configuration file, before you try the program again.

When the detection is finished, a path window will show.

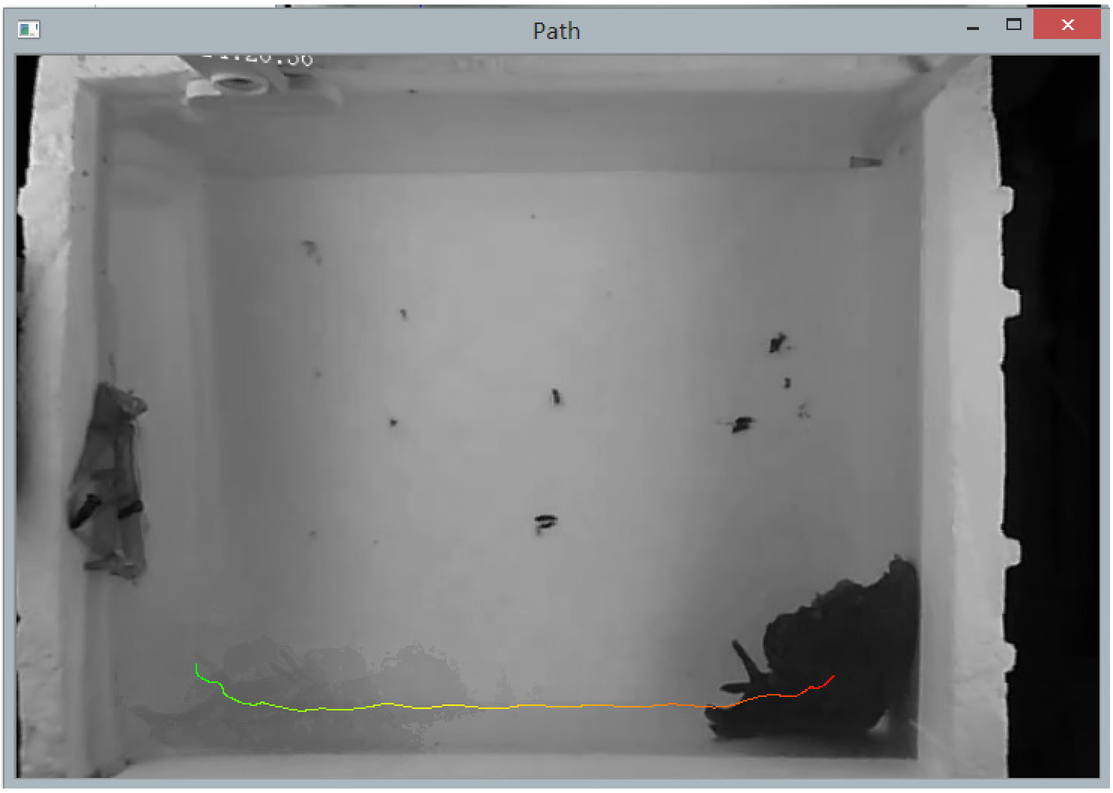

The red end of the path is the starting point, and the green end is the end point.

If the program is running correctly, the command line outputs will look something like this.

```
Cannot open alignfile, regenerate.
Calculating align...
Detecting...
Calculating path...
Length of path: 232.702 mm
```

The length of path and the coordinates of the animal from each frame detected will be in the result file.

4. Errors

(1) "Error: Cannot open video file."

Check if the directory for the video file is correct. Also note that *.mp4, *.avi and *.3gp are the compatible formats. If your video is not in these formats, change its format using format conversion tools.

(2) "Error: Invalid align file"

Delete the align file and try again.

5. Contact:

For further information, please contact: Chao-Yu Yang, poooon@foxmail.com

6. Appendix:

How to compile the source code:

1. Download and install CMake and MinGW.
2. Open cmake-gui, edit the textbox “Where is the source code” and “Where to build the binaries”, click **Configure**.

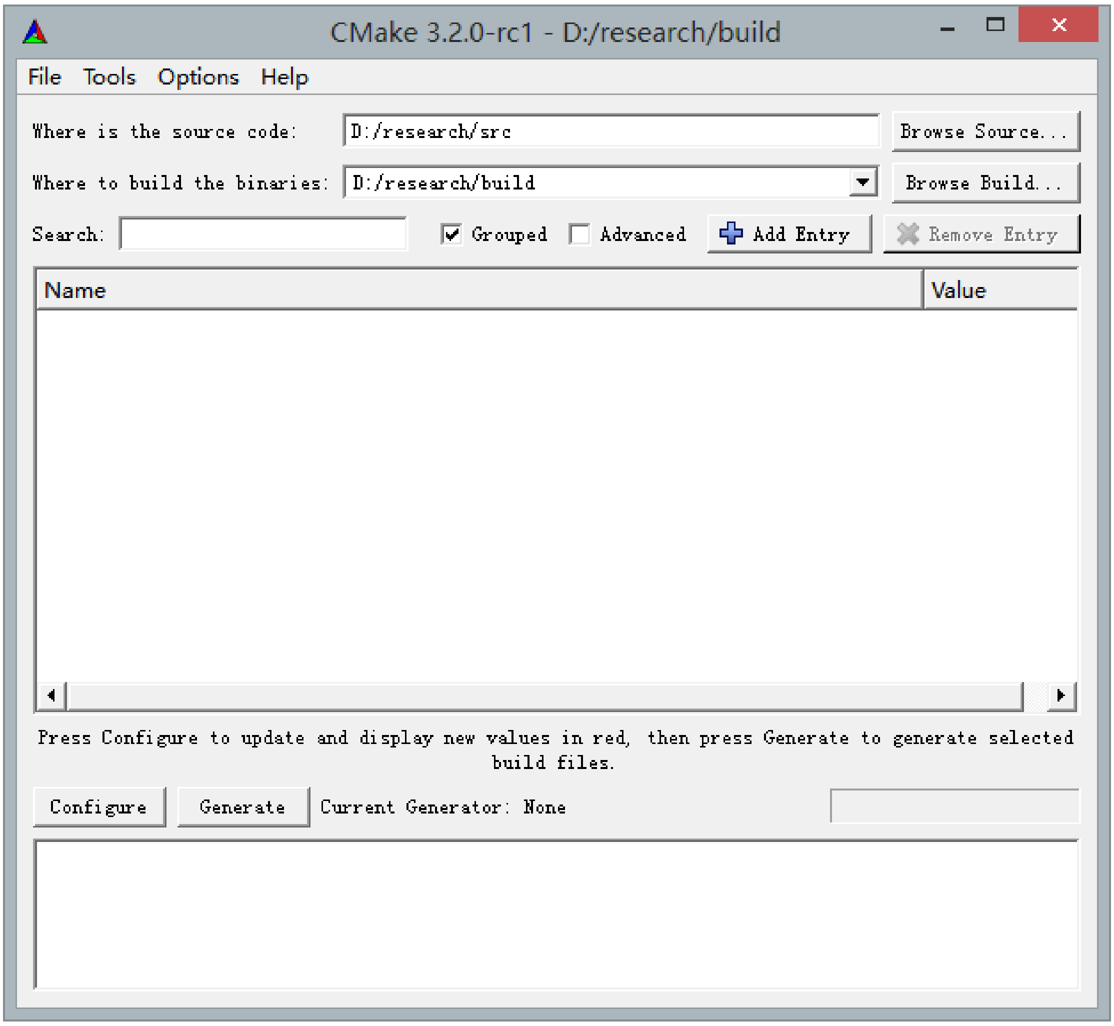

3. Select “MinGW Makefiles” and “Use default native compilers”, click **finish**.
4. If error(s) is shown at the bottom of the textbox, click “**File**”->”**Delete Cache**” and try again. If no error is shown, click **Generate**.
5. Open cmd, change directory to “Where to build the binaries” path, then execute “mingw32-make.exe” (which can be found in the installed MinGW folder) to start

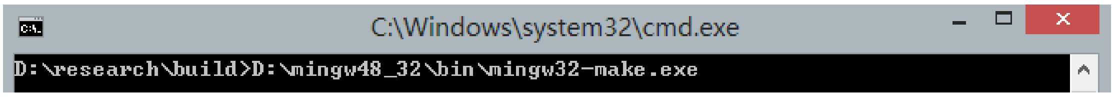

compiling.

6. When it is finished, find the executable file “analyzer.exe” in “Where to build the binaries” path, and replace the original one in the program folder.
