# Supplementary material for: Aplysia Locomotion: Network and Behavioral Actions of GdFFD, a D-Amino Acid-Containing Neuropeptide
Source: PLoS One. 2016 Jan 21;11(1):e0147335. doi: 10.1371/journal.pone.0147335 (PMC4721866; doi:10.1371/journal.pone.0147335)
Supplement: S2 Text — (PDF) [file pone.0147335.s002.pdf]

Supporting information 4

Validation of detection

During recording, camera could be blocked accidentally, so it is necessary to check if the detection of an object in a frame is valid. Our program uses two criteria to verify if the detection is valid:

1. Size of detected object area. The animals we used were in certain sizes (between 100-250 g). If the detected area was too large (above 15000 mm^2) or too small (below 1000 mm^2), or even did not exist, the frame was deemed invalid.
2. Distance between the center of the detected area of the frame and the previous valid one. Aplysia cannot move faster than 60 mm/s, and the noise-caused bias is smaller than 3 pixels, so the position of animal between neighboring valid frames should be smaller than 25 mm (calculated distance), which is set as the default limit. If the calculated distance between a valid frame and its next frame is bigger than the limit of 25 mm, the next frame was considered invalid. There could be several continuous invalid frames, so we calculated the distance between one frame to the previous valid one, and the default limit was multiplied by the number of invalid frames between them.

If a detected object in a frame is invalid, its position data will be ignored, and the lost data in this frame will be completed by interpolation as described below.

Interpolation in frame alignment and position data completion

We used interpolation in our program in two places: frame alignment and position data completion. We utilized liner interpolation algorithm in both cases. The principle for the algorithm is the following:

If we have an incomplete list of points $\{P_n\}$ and want to interpolate between the point P_a and point P_b $(b > a)$, whose position are (x_a, y_a) and (x_b, y_b), then we use $(x_a + (k - a)(x_b - x_a), y_a + (k - a)(y_b - y_a))$ as the interpolated point P_k between P_a and P_b.

In frame alignment, to reduce the amount of data needed to process, our program only uses one frame in every 100 frames to calculate alignment parameter, which is a 2D point that represents frame movement due to unexpected outside force in x and y directions, and interpolate other alignment parameters.

In position data completion, whenever there is an invalid data point, our program interpolates between every valid data with its next valid data. If there is no invalid data between them, nothing will be interpolated.
