# Supplementary material for: Aplysia Locomotion: Network and Behavioral Actions of GdFFD, a D-Amino Acid-Containing Neuropeptide
Source: PLoS One. 2016 Jan 21;11(1):e0147335. doi: 10.1371/journal.pone.0147335 (PMC4721866; doi:10.1371/journal.pone.0147335)
Supplement: S3 Text — (PDF) [file pone.0147335.s003.pdf]

Supporting information 5

Filtering

The filtering algorithm in our program uses average filtering, which is calculating the convolution of a signal (x or y data of position) and a rectangular function (see Figure at the end):

$$f(x) = \frac{1}{2w}[u(x+w) - u(x-w)]$$

"w" is the "half width" of the rectangular function. If the points in the non-filtered path are $P_n(x_n, y_n)$, the positions of every point $P'_n(x'_n, y'_n)$ in the filtered path can be calculated as:

$$x'_k = \sum_{i=-w}^{w} \frac{x_i}{w}$$

$$y'_k = \sum_{i=-w}^{w} \frac{y_i}{w}$$

Note that "w" of the rectangular function can significantly affect the length of the path that is being filtered. When w is small, the effect of filtering is insignificant, and the length of path does not shorten substantially. When w is big enough, the path will not decrease significantly further if w becomes bigger, and this is a sign that nearly all of noise has been filtered out. On the other hand, if we continue to increase w further, we may lose some details. Thus, our program sets $w = 100$ as the default, which can remove most of the noise without too many loses in details.

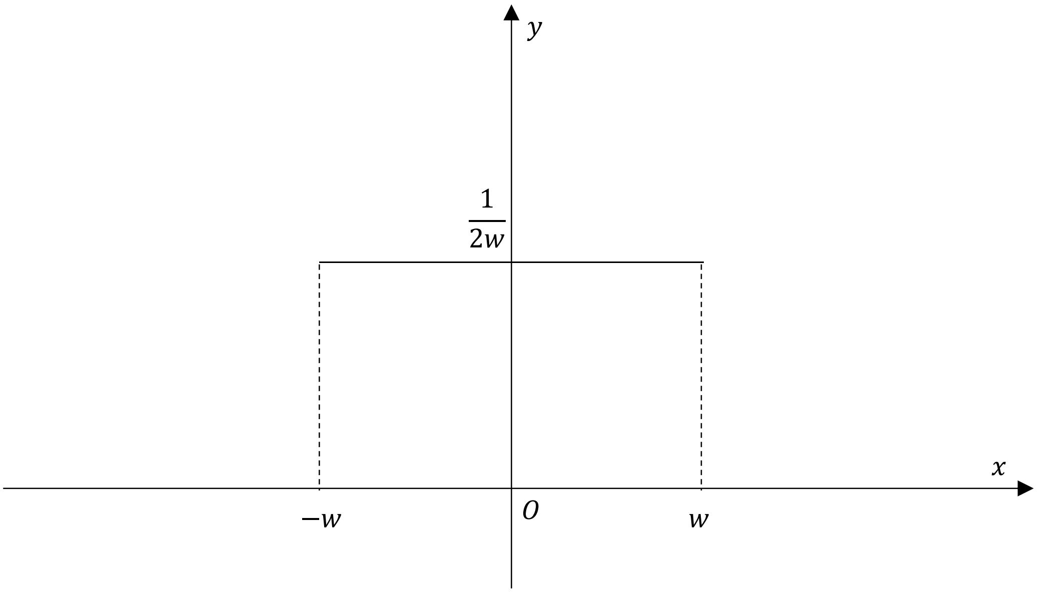

Fig. a rectangular function.
